# Buffalo Medical and Surgical Association

**Published:** 1883-05

**Authors:** Clayton M. Daniels

**Affiliations:** Secretary


### Society Reports.

### Buffalo Medical and Surgical Association.

*Regular Annual Meeting, April 3d, 1883.*

Vice-President, Dr. John Cronyn, in the Chair. Dr. C. M. Daniels, Secretary.

Present—Drs. Hauenstein, Bartlett, Coakley, Burghardt, Barker, Heath, Gay, Briggs, Brecht, Peterson, Stockton, Granger, Hubbell, Clark, Hopkins, Tremaine, Hartwig, Dorland, C. A. Ring, Wall, Mann, York, Strong, Bartow, Fowler, Moody, MacNiel, McBeth, Howe, Potter, Fell, and by invitation, Drs. Hayd and Crego.

Application for membership was received from Dr. F. S. Crego, who was duly elected.

*Essay*—By Dr. Frederick Peterson; subject, "Contagium Animatum." The paper was a comparatively full elucidation of the more recent ideas of the origin, development and transmission of disease. It was listened to with marked interest and called forth the following:

*Discussion*—Dr. Strong thought that much could be said, and alluded to the difficulties we all feel in trying to decide as to whether bacteria are the cause or effect. As for himself, he still remained in doubt.

Dr. Mann remarked that one point the essayist had evidently forgotten to mention, viz., the method by which the bacteria produce disease.

Alkaloid substances had been found in the excretions and supposed to be the product of the bacteria, and said that if large trees, etc., can produce an alkaloid, why is it not possible with these small fungi? It seems very puzzling that bacteria will infest the body and yet produce disease; we may suppose one of two things, either they are not the same as they appear, or a certain state of the system gives them a virulent property.

Dr. Howe said the subject was one of too much importance to pass without some comment. Referring to the great mortality from contagious diseases, he said it was given by authority that during the last ten years the number of deaths in the United Kingdom from "scarlatina" alone was ninety-two thousand, this being greater than the loss for the like period during the Napoleonic war. He believed that spontaneous disease did not occur; and speaking of the germs themselves, he thought it highly probable that although there might be a striking resemblance in their general appearance, in other properties they might be entirely different, and that we really had no right to say that, even though they swarm in the body, they are the same.

Dr. Hartwig thought the paper an excellent one, and believed that bacteria are the cause of more than was generally supposed, and especially referred to their action in pneumonia and acute rheumatism. He asked to be informed in regard to the observations of Tyndall upon the subject.

Dr. Fell was gratified that Dr. Peterson kept abreast of the times. He believed in Koch and his discoveries, as his mistakes were rare; also that disease was caused by these minute organisms; he considered Tyndall's experiment a success in its revelation of these germs, and that we are on the way to know more of these germs in their causation of disease.

Dr. Barker believed in the theory, that possibly all diseases were caused in this way; he spoke of the bacteria in the cocoon which was so destructive to the silk interests several years ago, and that it was discovered by scientific investigation and successfully combatted. He thought it compared favorably with the recent discoveries of Koch and others, and that the diseases would be provided with a successful remedy.

Dr. Barrett spoke of the rapidity of reproduction and development of these organisms. He believed they infected only under certain conditions, and compared it to certain fungus growths upon grain which are only developed when the condition became favorable.

Dr. Tremaine spoke of the antiseptics used to destroy the germs, and the resisting power of normal healthy tissue, and in surgery, that granulating wounds were less likely to receive poisonous germs.

Dr. Hopkins felt under personal obligations to the essayist, and believed in the long stated chemical and germ theories, and that they might be found to act together. Also that possibly any chemical substance might infuse a deleterious influence. Possibly all fermentation may be the excretion or secretion of the fungus plant.

Dr. Bartlett remarked that if the germ theory is not right, what is? It seemed that we were irresistibly drawn to it, and that the rise of temperature was caused by fermentation. He thought the spread of certain zymotic diseases might depend upon a special pabulum which once destroyed was not renewed, thus exempting the individual from further attacks. A modification, also, by cultivated views might deprive it of that quality which sustains special spores or bacteria.

Dr. Hayd spoke of the necessity for scientific investigation and general treatment of disease. So far, it was acknowledged that each produced its like; referred especially to his observations while in Vienna, that substances such as hay infusion, after passing through a fermentive process and exposed to the air, seemed to take up new and virulent organisms which were proven poisonous, as anthrax.

Dr. Cronyn said the application to prove its value in medicine and surgery was the great point, and that the same appearing germs produce different affections. To his mind, it was in the condition of the individual, and made but little difference which brought the influence to bear upon the sympathetic centers.

Dr. Peterson in closing said he tried to prove the necessity for a living contagium; it was generally accepted that it was a growing poison and that it must be organic. In reply to Dr. Hartwig, he said that Tyndall's observations were made by passing an electric beam through the ice of distilled water.

The regular order of business was suspended.

The Secretary read the following report:

As your Secretary for the past year, I take pleasure in reporting that it has been one of exceptional progress, the largely increased attendance being an average of *twenty-four* as compared with only *ten* for several preceding years, together with the interesting and instructive character of the several papers on a diversity of subjects, has awakened a decided interest in the proceedings of this society. New life and vigor seemed to be infused, and the efforts of those interested have been warmly seconded and encouraged.

Our membership has now reached a total of eighty-four, having received an addition of seventeen during the past year.

The publication of the monthly proceedings has doubtless been an important factor in aiding our advancement, and to the JOURNAL we at least owe a full appreciation.

The removal to our present location seems to have been a wise procedure, as it is far more convenient for a large number of members.

Financially, the association is in a highly prosperous condition, the annual dues and fees received from new members being ample for all necessary expenses.

Although it has been necessary to drop several names from our list of membership on account of non-payment of dues, the act has had but a wholesome effect and insured greater promptness for the future.

The society never had better prospects, and it is sincerely to be hoped that all will feel a professional pride in trying to promote its prosperity.

CLAYTON M. DANIELS, *Secretary.*

The Treasurer also reported as follows:

| | |
|---|---|
| Cash on hand April 4, 1882, . . . . . . . . | $58.74 |
| Received during past year, . . . . . . . . | 77.25 |
| | $135.99 |
| Disbursements, . . . . . . . . . . . . . | 89.65 |
| Balance on hand, . . . . . . . . . . . . | $46.34 |

F. E. L. BRECHT, *Treasurer.*

Drs. Fowler and Barker were appointed an Auditing Committee.

The election of officers for the ensuing year being in order, Dr. Hopkins moved that a nominating committee be appointed.

Dr. Howe vigorously opposed the motion as being a bad precedent, as it had been attempted once before and was productive of unpleasant results.

Dr. Hopkins then withdrew his motion.

The election resulted in the following:

President, John Cronyn; Vice-President, F. W. Bartlett; Secretary, Clayton M. Daniels; Treasurer, F. E. L. Brecht; Librarian, Jas. B. Samo.

The Auditing Committee reported the accounts of the Treas urer correct. And the meeting adjourned.

CLAYTON M. DANIELS, *Secretary.*

